# Toxicity and Anticancer Potential of *Karwinskia*: A Review

**DOI:** 10.3390/molecules25235590

**Published:** 2020-11-27

**Authors:** Gilberto Jaramillo-Rangel, María-de-Lourdes Chávez-Briones, Alberto Niderhauser-García, Marta Ortega-Martínez

**Affiliations:** Department of Pathology, School of Medicine, Autonomous University of Nuevo Leon, Monterrey 64460, Mexico; gjaramillorangel@yahoo.com.mx (G.J.-R.); mdlcb.71@gmail.com (M.-d.-L.C.-B.); a_nider_garcia@yahoo.com.mx (A.N.-G.)

**Keywords:** *Karwinskia*, peroxisomicine A1, anticancer agent, natural bioactive compounds, activity of plant extracts

## Abstract

*Karwinskia* genus consists of shrubs and small trees. Four toxic compounds have been isolated from *Karwinskia* plants, which were typified as dimeric anthracenones and named T496, T514, T516, and T544. Moreover, several related compounds have been isolated and characterized. Here we review the toxicity of the fruit of *Karwinskia* plants when ingested (accidentally or experimentally), as well as the toxicity of its isolated compounds. Additionally, we analyze the probable antineoplastic effect of T514. Toxins cause damage mainly to nervous system, liver, lung, and kidney. The pathophysiological mechanism has not been fully understood but includes metabolic and structural alterations that can lead cells to apoptosis or necrosis. T514 has shown selective toxicity in vitro against human cancer cells. T514 causes selective and irreversible damage to peroxisomes; for this reason, it was renamed peroxisomicine A1 (PA1). Since a significant number of malignant cell types contain fewer peroxisomes than normal cells, tumor cells would be more easily destroyed by PA1 than healthy cells. Inhibition of topoisomerase II has also been suggested to play a role in the effect of PA1 on malignant cells. More research is needed, but the evidence obtained so far indicates that PA1 could be an effective anticancer agent.

## 1. Introduction

The genus *Karwinskia* (*Rhamnaceae* family) consists of small trees and shrubs native to southern United States and Mexico in North America; Guatemala, El Salvador, Honduras, Nicaragua, Costa Rica, and Panama in Central America; Colombia and Venezuela in South America; and Cuba, Haiti, and the Dominican Republic in the Caribbean [1,2].

There are 22 accepted species of *Karwinskia* (Table 1, [2,3]). Species are often spined; leaves are simple, opposite or nearly so, usually stipulate, entire or toothed; flowers are greenish or yellowish grouped in short-stemmed clusters in the axils of the leaves; the fruit is capsular or drupaceous, green when immature turns dark purple on ripening; the wood is hard and strong [1,2,4]. *K. humboldtiana* is the most widespread and studied of the species, and is also known as tullidora, cacachila, coyotillo, tuvii, capulin, wild cherry, and pimientillo, among other names [5].

Toxicity of the fruit of *K. humboldtiana* was known by indigenous tribes before the arrival of Europeans to America [6]. It was not until 1789 that Clavigero described the poisonous properties of *K. humboldtiana*. He wrote in his book “Historia de la Antigua o Baja California” that native children suffered paralysis after eating the fruit, and in some cases death occurred [7]. Castillo-Najera reported a similar intoxication in a troop of 106 soldiers during the Mexican revolution, with a mortality rate of over 20%. Autopsies indicated peripheral nerve damage [8]. Standley stated that seeds of *K. humboldtiana* contain some principle that paralyzes the motor nerves [9]. In a series of classical studies published in 1928, Marsh et al. showed that the fruit of *K. humboldtiana* was toxic to several animal species, in which alterations were observed not only in the nervous system, but also in organs such as liver, kidney and lung [4]. Subsequently, cases of poisoning continued to be reported, but the biological causes of the observed alterations were still unknown [10].

In 1975, Dreyer et al. [11] isolated for the first time four toxic principles from the seed of *K. humboldtiana*. Interestingly, one of them has also shown antineoplastic potential.

## 2. Compounds Isolated from the *Karwinskia* Genus

**Figure 1:**
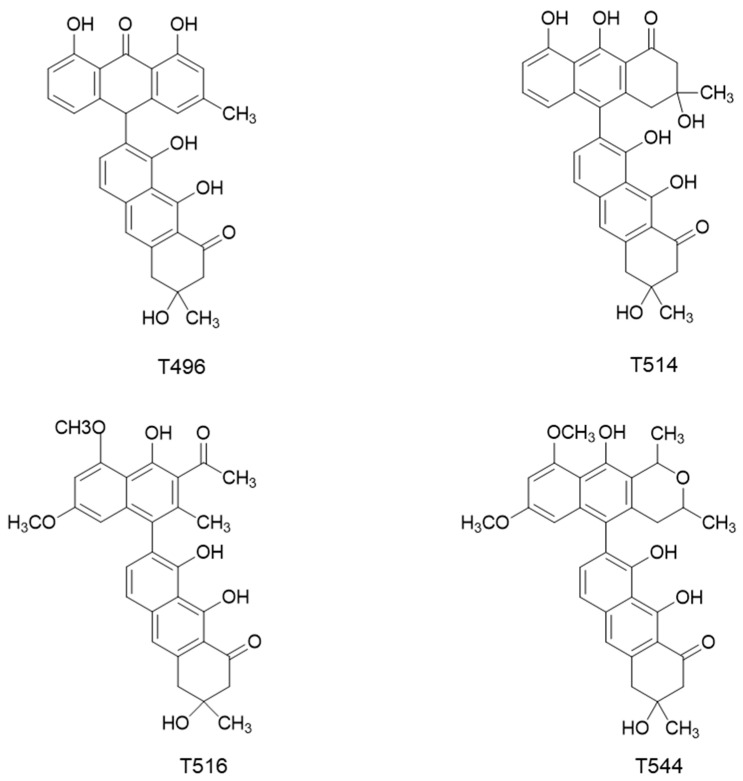
Chemical structures of the four toxins isolated from *K. humboldtiana*.

As mentioned above, Dreyer et al. [11] isolated and characterized from the seed of *K. humboldtiana* four toxins, which were typified as dimeric anthracenones. According to their molecular weight, these molecules were called T496, T514, T516, and T544 (Figure 1). Waksman et al. [12] demonstrated later that these toxins are characteristic of the plants from *Karwinskia* genus. Guerrero et al. [13] published a modification of the method described by Dreyer et al. in order to simplify the procedure of purification of T496, T514, and T544.

Toxicological studies performed in mice demonstrated that T514 and T544 causes neurological damage, besides alterations in organs such as liver, lung, and kidney [14]. In vitro studies described for the first time a selective toxicity of T514 on human tumor cells [15]. At sublethal doses, T514 produces an irreversible and selective damage of yeast peroxisomes in vivo [16]. For this reason, T514 was renamed peroxisomicine.

Some isomers of T514 or peroxisomicine have been isolated. The letter A was added to the “peroxisomicine” name due to the Cotton effect that shows in curves obtained by circular dichroism (CD) spectroscopy and was adopted for the nomenclature proposed for similar compounds [17,18]. Additionally, for being the first isomer isolated, the number 1 was added to the name, turning it to be peroxisomicine A1 (PA1). Waksman and Ramírez-Durón [18] isolated from fruits of *K. parvifolia* a stereoisomer which gave the same effect in the CD curve and was named peroxisomicine A2 (PA2). Through X-ray analysis, relative stereochemistry of PA1 and PA2 was established: in PA1 carbons C-3 and C-3′ have the same stereochemistry, whereas in PA2 these carbons have opposite stereochemistry [19,20].

**Figure 2:**
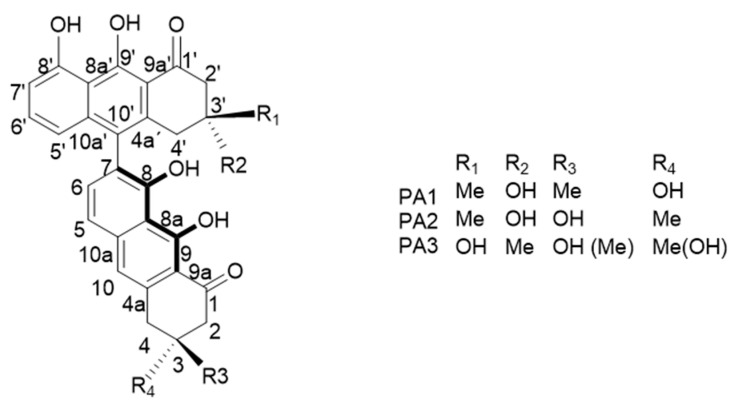
Isomers of T514 (peroxisomicine A1 or PA1).

Other isomers have also been isolated from *K. parvifolia*. A third compound was named peroxisomicine A3 (PA3). The CD curve for PA3 exhibited the same effect that PA1 and PA2. UV (ultraviolet), ^1^H-nuclear magnetic resonance (NMR), and ^13^C-NMR spectra of PA3 were very similar or identical to those exhibited by PA1 and PA2. Thus, it was concluded that the planar structure of PA3 is the same as that described for PA1 and PA2. Examination of ^1^H and ^13^C spectra using heteronuclear multiple quantum coherence (HMQC) and heteronuclear multiple bond coherence (HMBC) with different pulse lengths led to the assignment of the structures of PA1, PA2, and PA3 shown in Figure 2 [6,20].

**Figure 3:**
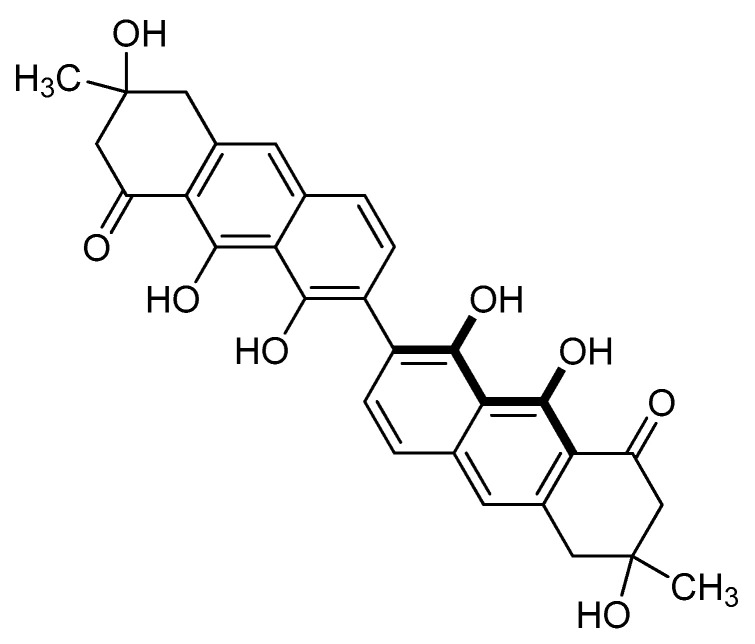
Chemical structure of Isoperoxisomicines.

Two other compounds having a molecular weight of 514 were isolated. UV-Vis (visible) spectra indicated that the chromophores were similar to those present in peroxisomicines. In ^1^H- and ^13^C-NMR spectra, these compounds showed the half of the expected signals, according to their molecular weight. This simplicity of NMR spectra suggested a symmetrical structure for both compounds. From ^1^H-NMR spectra, a C-7, C-7′ linkage between the naphthalene subunits was proposed. Complete assignment of all carbons signals was carried out by means of HMBC and HMQC experiments. CD curves were similar for both compounds and showed the same effect than peroxisomicines. The compounds were named isoperoxisomicines A1 and A2 (Figure 3). Although their absolute stereochemistry remains undefined, according to NMR spectra one should be the 3R,3′R isomer and the other the 3S,3′S isomer [6,20]. Isolation of isoperoxisomicine A1 from fruits of *K. humboldtiana* has also been reported [21].

Garza-Ocañas compared the hepatotoxicity of PA1 (T514) and PA2 in primary liver cell cultures. Toxicity was evaluated by release of lactate dehydrogenase (LDH), and mitochondrial metabolic function (reduction of 3-[4,5-dimethylthiazol-2-yl]-2,5-dephenyltetrazolium bromide, MTT). The in vitro hepatotoxicity of PA2 was very similar to that of PA1 [22]. In vivo, animals treated with PA2 showed a lesser grade of lesions in liver, lung, and kidney in comparison with the PA1 treated ones [23]. On the other hand, all isomers of T514 (PA2, PA3, and isoperoxisomicines A1 and A2) showed toxicity against hepatoma cell lines [20].

It is necessary to know exactly the composition and quantification of compounds present in the substances used in biological tests like those described in the later paragraph. Various High Performance Liquid Chromatography (HPLC) methods applicable to the substances isolated from *Karwinskia* have been developed: Salazar et al. [24] described a method applicable to PA1, PA2, T516, and T496; Bovanová et al. [25] described a method for PA1; and Osorio-Pérez et al. [26] developed another to analyze PA1, PA2, PA3, and isoperoxisomicines A1 and A2.

**Figure 4:**
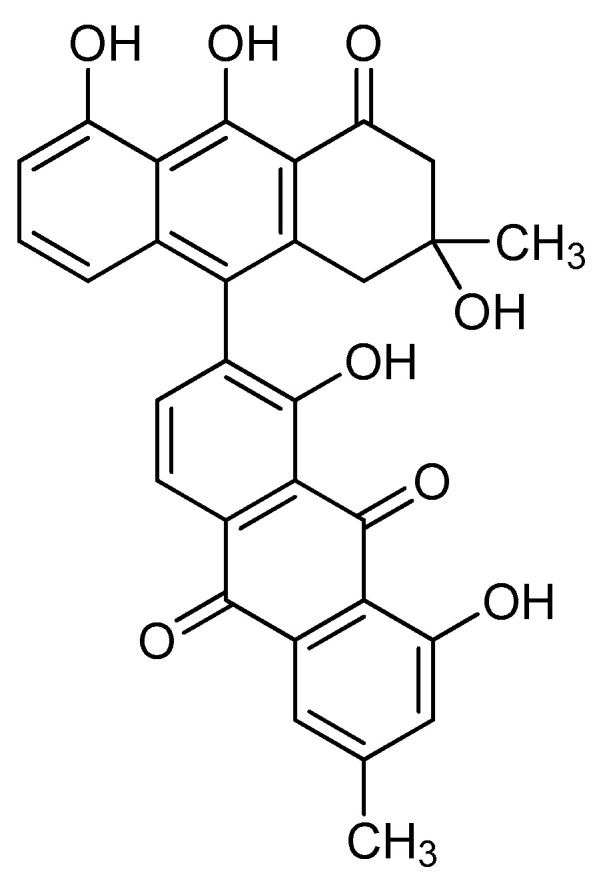
Chemical structure of T510.

Rivas et al. [21] isolated from the fruit of *K. tehuacana* T496, T514, and T544, but also a fourth compound characterized by chromatography and spectra data as anhydroperoxisomicine-quinone-A1 or T510 (Figure 4). Researchers were not able to find even traces of T510 after analyzing 50 samples of *K. humboldtiana* collected in different locations, which suggest that the isolation of T510 can be used to distinguish between the *tehuacana* and the *humboldtiana* species. T510 showed similar toxicity than T514 in preliminary studies [21].

Waksman et al. [27] isolated from air-dried ground roots of *K. parvifolia* two compounds which are isomers with the same planar structure of T544. The identification methods were similar to those described above for the other compounds. Although more experiments are needed in order to fully establish the stereochemistry, existing data indicate that both isomers are epimers, with differences in the chirality of C-3. Sometimes T544 is called tullidinol, and thus, these isomers were named tullidinol B1 and B2. Letter B indicates the Cotton effect that both compounds showed in CD curves, and the numbers 1 and 2 correspond to the order of their isolation by HPLC. Tullidinol B1 and B2 were found to be biologically active (*A. salina* test). Interestingly, these compounds are not present in the fruits of *K. parvifolia*, while T514 and T496, which are abundant in the fruit, are absent in the roots. This feature appears to be unique to *K. parvifolia* [18,28].

Finally, other related compounds have been isolated from plants of the *Karwinskia* genus. Mitscher et al. [29] isolated from the roots of *K. humboldtiana* three constituents: karwinaphthol A, karwinaphthol B, and 2-acetyl-6,8dimethoxy-3-methyl-1-naphthol. These compounds showed some antimicrobial activity. Yussim et al. [30] isolated from the roots of *K. humboldtiana*, *K. johnstonii*, *K. mollis*, *K. subcordata*, and *K. umbellata* two anthraquinones and the 7′desmethoxy analogue of T514. No biological activity was reported for these compounds. Rojas-Flores et al. [31] isolated from the fruits of *K. parvifolia* two dimeric dihydroxyanthracenones, karwinaphthopyranones A1 and A2, and three dimeric dihydroxyanthracenones with different oxidation states, karwinaphthopyranones B1, B2, and B3. Karwinaphthopyranone B1 showed antiproliferative activity against the human cancer cell lines Hep G2 (liver), ZR-7530 (breast), and HCT-15 (colon). Ramírez-Durón et al. [32] carried out derivatization procedures on the phenolic OH groups, carbonyl group, and aromatic ring of T514 (PA1). Six new compounds were obtained and characterized. The selective cytotoxicity of the compounds on both normal and cancerous human liver cells lines was evaluated. Cytotoxicity assays suggested that phenolic groups are necessary for the biological action of PA1.

In summary, the main bioactive agents of *Karwinskia* plants have been isolated and characterized, including toxins T496, T514, T516, and T544, and several related compounds, such as peroxisomicine A2, peroxisomicine A3, isoperoxisomicines A1 and A2, toxin T510, and tullidinol B1 and B2.

In this article, we review the toxicity of the fruit of plants of the genus *Karwinskia* when ingested (accidentally or experimentally), as well as the toxicity of its isolated compounds. In addition, we review the probable antineoplastic effect of T514. Literature on the probable anticancer properties of *Karwinskia* refers almost exclusively to this compound.

## 3. Damage Caused by *Karwinskia*

### 3.1. Clinical Aspects

Since the first cases described by Clavigero and Castillo-Nájera ([7,8]), numerous cases of intoxication in humans and animals due to accidental ingestion of the fruit of *K. humboldtiana* have been reported in the literature [10,33,34,35,36,37,38,39]. Poisoning with this plant may represents a public health problem in certain geographical areas. In Mexico, Nava et al. [40] established three risk areas: the southwestern central part, the arid northern area, and the arid and dry central area. Most of the analyzed cases were from communities with low levels of education and low socioeconomic conditions. It has been estimated that in Mexico there are between 30 and 40 human deaths a year caused by *K. humboldtiana* [41].

The severity of the disease that develops in humans and animals after accidental ingestion of *K. humboldtiana* fruit is proportional to the amount of fruit ingested. High dose fruit ingestion (acute intoxication) causes death after 24–48 h by cardiopulmonary arrest without paralysis signs, with extensive damage in lungs, liver and kidneys [14,42,43]. If the fruit is consumed in small quantities but chronically (chronic intoxication), produces 2–3 weeks later a distal, ascending, progressive flaccid paralysis of the limbs, clinically similar to Guillain-Barré syndrome, poliomyelitis, and other polyradiculoneuritis syndromes. In severe cases, paralysis may progress to quadriplegia, respiratory failure, bulbar palsy, and even death. In other cases, the damage can be reversed with physical and symptomatic therapy towards a full recovery in 6 to 12 months [33,35,36,37,38,39,44,45].

Clinical diagnosis of intoxication with *K. humboldtiana* fruit may be very difficult if there is no prior evidence of ingestion of the fruit. Most patients present with nonspecific signs and symptoms, including diarrhea, vomiting, loss of strength in limbs, breathing difficulty, fever, coughing, and myalgias. The latency period between ingestion and onset of nervous symptoms also difficulties diagnosis. Moreover, intoxication can be interpreted as Guillain-Barré syndrome, poliomyelitis, or other similar pathologies [10,33,34,35,36,37,38,39]. With respect to the diagnosis methods, peripheral polyneuropathy with segmental demyelinization is a hallmark of electrodiagnostic studies. Motor conduction studies are abnormal, with a decrease in velocity of conduction of 40% to 60% with respect to normal values. Sural nerve biopsy shows segmental demyelinization without inflammatory infiltrate. Cerebrospinal fluid does not reveal abnormalities [46,47,48,49]. Otero et al. [50] detected T544 in the blood of toxin-treated rats using spectrophotometric and chromatographic methods. The presence of toxins in the blood of patients intoxicated by *K. humboldtiana* can be determined by thin-layer chromatography (TLC) [38,41,51].

At the present time, the mechanism of damage caused by *K. humboldtiana* toxins is not fully understood. Therefore, there is no specific antidote for these toxins. Treatment consists of supportive care and physical rehabilitation [47,49,51]. Free radicals have been associated to demyelinization observed in certain types of neuropathies, including diabetic neuropathy [52]. Furthermore, oxidative stress may be a mechanism of toxicity of T514 (see below). For these reasons, García-Juárez et al. [53] administrated α-lipoic acid, a powerful antioxidant, to rats intoxicated with the fruit of *K. humboldtiana*. However, no improvement was observed on the clinical manifestations or in the histopathological lesions evaluated. It has been suggested that administration of thiamine may be helpful [54].

### 3.2. Damage to Nervous System

#### 3.2.1. Peripheral Nervous System (PNS)

Most of the published reports about *Karwinskia* toxicity are focused on the PNS. Oral administration of crude homogenate of *K. humboldtiana* fruit, or toxins isolated from it, produces distal segmental demyelinization and degenerative changes of peripheral nerves in several animal species [14,55,56,57,58,59]. In turn, these changes produce a conduction block of nerve impulses [60,61] and a depression of fast axonal transport [62,63,64]. Furthermore, it has been observed that distal hind limb muscles of treated animals are partially denervated [60,65].

Axonal degeneration is more extensive at the ends of long nerves [55,56,57]. Regeneration may occur in nerve fibers which have undergone degeneration [55,59].

Histopathologically, Bermudez et al. [14] found in mice treated with either the fruit of *K. humboldtiana* or toxins T514 or T544, swollen nerve fibers, disorganized and vacuolated myelin sheaths, and areas of segmental demyelinization, without inflammatory infiltrate. Similar findings have been encountered in intoxicated humans [39,51].

It has been proposed that Schwann cells are the primary target of *Karwinskia* toxins. Charlton and Pierce [55,56,57] reported an increase in the cytoplasmic volume of Schwann cells in damaged nerve fibers in goats experimentally intoxicated with the fruit of *K. humboldtiana*. Ultrastructurally, Schwann cells showed degeneration of mitochondria and glycogen depletion. Mitochondrial injury would result in impaired active transport and in the intracellular edema previously described [57]. In fact, in vitro studies demonstrated that extracts of the fruit of *K. humboldtiana* cause inhibition and uncoupling on respiration and oxidative phosphorylation of mitochondria [66]. In vivo studies showed that intoxication with *K. humboldtiana* seed decrease the ATP synthesis [67] and produces alterations in membrane fluidity and ATPase activity in submitochondrial particles [68]. These findings suggest an important role of mitochondria in the mechanism of action of *Karwinskia* toxins. Additionally, Mitchell et al. [69] injected T496 and T544 dissolved in sesame oil directly into the sciatic nerve of rats. Oil droplets were observed in the cytoplasm of Schwann cells, which suggest that the toxins have a primary action on these cells. 

On the other hand, there is evidence of a direct action of *Karwinskia* toxins on the neuronal elements. Aoki and Muñoz-Martinez [58] suggested that toxins may be taken up by the motor nerve terminals, perhaps by endocytosis, and thus would be more concentrate in these sites. Also, Heath et al. [70] using myelinated organotypic cultures of nervous tissue exposed to T496 and T544 found a primary effect upon axons, mainly a widening of the periaxonal space and a redistribution of axonal organelles leaving central regions occupied by neurofilaments. These changes preceded axonal degeneration. 

However, Muñoz-Martínez et al. [64] found alterations in both Schwann cells and axons into the right sciatic nerve in cats injected with T544 and concluded that both structures are T544 targets. Thus, more research is necessary in this area.

#### 3.2.2. Central Nervous System (CNS)

Charlton and Pierce [71] reported that an axonal dystrophy occurred in the CNS of goats experimentally poisoned with the fruit of *K. humboldtiana*. Lesions were more evident in the cerebellum (especially in the neocerebellum), while some swelling occurred in axons of the white matter of the spinal cord. There was a positive relationship between the cerebellar lesions and clinical signs observed in affectated goats: increased alertness, tremor, increased responsiveness to external stimuli, and high-stepping movements.

Ortiz et al. [72] studied the cerebral motor cortex, the CA1 region of hippocampus, and the caudate nucleus in rats orally intoxicated with the fruit of *K. humboldtiana*. Hyperchromasia, cell death, and widening of the Virchow-Robin spaces were observed in cerebral motor cortex; hyperchromasia, cell shrinkage, and cell death were evident in the CA1 region of hippocampus; and neuronphagia and cell shrinkage occurred in caudate nucleus. These alterations may be related to the nonparalytic motor disturbances occurring early after *K. humboldtiana* intoxication.

In rats chronically poisoned with the fruit of *K. humboldtiana*, Becerra-Verdin et al. [73] found chromatolysis in most neurons of the pons nuclei. The corticopontocerebellar tracts presented alterations, which probably caused the chromatolysis seen in their corresponding neurons. Lesions in the pons may cause the equilibrium loss observed before paralysis in intoxicated rats. On the other hand, a loss of Purkinje cells, or alterations in their morphology (retraction, hyperchromasia, pyknosis) were found in some areas of the cerebellar cortex. The terminal deoxynucleotidyl transferase dUTP nick end labeling (TUNEL) reaction was negative, therefore the loss of neurons was not caused by apoptosis. Damage to Purkinje cells causes movement alterations, as those seen in intoxication with *K. humboldtiana*. Finally, in the cerebral motor cortex, pyramidal internal and external neurons showed characteristics related to ischemia and hypoxia.

Using the same experimental model mentioned in the last paragraph, Díaz-Pérez et al. [74] analyzed the histopathological alterations in the striatum. These authors found hyperchromic neurons, gliosis, and disorganization of the myelin sheaths and neuropil, along with increased axonal diameters. Changes observed may be related to the gait alterations, the weakness, and the paralysis observed in the intoxication caused by *K. humboldtiana*.

### 3.3. Damage to Liver, Lung, and Kidney

As mentioned earlier, copious ingestion of *K. humboldtiana* fruit causes death within first 48 h by cardiopulmonary arrest without paralysis signs, with extensive damage in several organs, mainly liver, lungs and kidneys [14,42,43].

Acute experimental intoxication of mice with either fruit or isolated toxins T514 or T544 of *K. humboldtiana* produced gross alterations in liver and lung. In liver, alterations consisted of degeneration of hepatocytes, necrosis of central zone, acute diffuse necrosis, congestion, and hemorrhage. In lungs, vascular congestion and hemorrhage were the principal findings [14]. 

A single oral preparation of *K. humboldtiana* fruit was given to rats, guinea-pigs, hamsters, and dogs. Interestingly, there were no clinical or histopathological evidence of damage in dogs. There is currently no explanation for this finding. Curiously, the coyote (*Canis latrans*), a species close to the dog, can eat the fruit of *K. humboldtiana* without becoming intoxicated. In fact, one of the common names for the plant is coyotillo [6]. All other species presented the same histopathological pattern. Liver showed sinusoidal congestion and centrilobular necrosis; massive necrosis occurred in some animals. Lungs showed interstitial congestion, edema, and massive hemorrhage. Kidneys depicted interstitial congestion and areas of cloudy swelling of the proximal convoluted tubules. Lesions in the liver and lung were considered as the cause of death of the intoxicated animals [42].

Several studies have attempted to clarify the mechanisms behind the histopathological findings described above. Garza-Ocañas et al. [75] assessed the cytotoxicity of T514 and T544 in primary cultures of rat hepatocytes and keratinocytes. Cytotoxicity was determined by release of LDH (plasma membrane integrity), MTT reduction (mitochondrial metabolic activity), and neutral red (NR) uptake (lysosomal activity). Hepatocytes were more sensitive to both toxins than keratinocytes. T514 was more hepatotoxic than T544. This finding is correlated with in vivo studies, in which T514 was more toxic to liver than T544 [14].

Production of reactive oxygen species (ROS) by T514 was analyzed in primary liver cell cultures and microsomes. The protective role of two antioxidant enzymes, catalase (CAT) and superoxide dismutase (SOD), against T514 cytotoxicity was also investigated. Cytotoxicity was determined by the MTT assay. The dichlorofluorescein diacetate (DCFDA) fluorescent probe and the reduction of ferricytochrome c were used as indicators of intracellular ROS. T514 induced the production of ROS in both hepatocytes and microsomes. CAT and SOD had a protective effect in both in vitro systems. It was concluded that T514 leads to the formation of ROS, and that oxidative stress may be a mechanism of cytotoxicity of T514 [76].

T514 injected intraperitoneally induced apoptosis in hepatocytes from mouse. Apoptosis occurred through the intrinsic mitochondrial apoptotic pathway. An increased expression of the pro-apoptotic markers p53, Bax, and Bak was observed, whereas a down regulation of the proliferating cell nuclear antigen (PCNA) and the anti-apoptotic molecule Bcl-2 occurred [77].

A single oral dose of ground *K. humboldtiana* seeds administered to rats resulted in a decrease in blood pressure, along with a marked reduction in renal perfusion pressure. Also, decrements of the glomerular filtration rate (GFR), renal plasma flow (RPF), and filtration fraction (FF), and an increment in the fractional excretion of sodium (FENa) were observed. These data suggest that the effect of *K. humboldtiana* in kidney is both on hemodynamic and epithelial parameters, since GFR, RPF, and FF are dependent on circulatory conditions, whereas FENa is mainly dependent on tubular epithelial function [43].

Using the same experimental model mentioned in the last paragraph, the effect of *K. humboldtiana* on kidney energetic metabolism was analyzed. In treated rats, the concentration of total proteins in renal tissue and the ATP concentration in renal cortex decreased significantly. Also, concentration of ATP in total blood and hemoglobin decreased significantly. These metabolic alterations might explain, at least partially, the mortality observed in the treated rats [67].

On the other hand, in the liver of rats chronically poisoned with the fruit of *K. humboldtiana*, García-Garza et al. [78] found necrotic areas, vascular congestion, and vacuoles in the hepatocytes. These pathological findings increased during intoxication phase and decreased in the recovery stage. Thus, in chronic intoxication with *K. humboldtiana* there is a damage in liver that may be reversible. In experimental chronic intoxication, damage in lung and kidney has also been observed. In lung, García et al. [79] found vascular congestion, fibrosis, and thickening of the alveolar septa by infiltration of mast cells. In kidney, blood vessel congestion, tubular necrosis and fibrosis of renal capsule were observed by light microscopy, while a thickening of the filtration barrier and of renal capsule was identified by electron microscopy [80].

### 3.4. Damage to Other Tissues and Organs

Dewan et al. [81] described degeneration of cardiac and skeletal muscles in goats caused by experimental feeding with *K. humboldtiana* fruit. In heart, some of the animals showed fatty degeneration involving all cardiac fibers, while other exhibited only areas of granular degeneration. Some fibers showed more advanced changes, like a greater eosinophilia of the sarcoplasm, or fragmented sarcoplasm with pyknotic nuclei. In skeletal muscles, alterations were more variable in severity, including vacuolar degeneration, sarcoplasm fragmentation, and proliferation of sarcolemmal nuclei with infiltration of macrophages and lymphocytes.

Carcano-Diaz et al. [82] examined the effects of acute intoxication with the *K. humboldtiana* fruit on pancreatic tissue of rat. In the exocrine pancreas, damage included a reduction in the number of zymogen granules, presence of autophagy-like vesicles, apoptosis, necrosis, and inflammatory infiltrate, culminating with a complete loss of acinar and lobular architecture. Of note, morphology of the islets of Langerhans was preserved.

### 3.5. Damage to Embryo and Fetus

Mouse embryos were exposed in vitro to T544 for 24 h. Alterations observed included encephalic dilatation, swelling and hemorrhage of the mandible, and kinky tail. Furthermore, a significant decrease in protein content was observed [83].

Later, the effect of *K. humboldtiana* toxins on the developing mouse embryo was analyzed in vivo. At day 8 of gestation, dams were injected intraperitoneally with either T514 or T544. Animals treated with T544 showed a significantly higher incidence of reabsorptions and malformations compared with control groups. Also, fetal crown-rump (CR) length and weight were significantly lower than controls. Malformations observed included lower limb deformities, abdominal evisceration, and exencephaly. The animals treated with T514 did not show significant differences compared with the controls. Thus, T514 had no teratogenic effects, which is important in view of its probable use as antineoplastic agent [84].

In summary, toxins of *Karwinskia* cause damage mainly to nervous system, liver, lung, kidney, cardiac and skeletal muscles, pancreas, and to embryo and fetus. The pathophysiological mechanism has not been fully understood but includes metabolic and structural alterations that can lead cells to apoptosis or necrosis.

## 4. *Karwinskia* as a Potential Antineoplastic Agent

Piñeyro-López et al. [15] analyzed the in vitro effect of T514 upon normal and neoplastic cells derived from liver (hepatoma), lung (adenocarcinoma, undifferentiated bronchogenic carcinoma, squamous cancer cells, small cell carcinoma) and colon (adenocarcinoma) of human origin, and compared the results with those obtained with the known antineoplastic drugs doxorubicin, epidoxorubicin, vincristine, 5-fluorouracil, and mitomycin. For T514, the minimum cytotoxic concentrations (MICC) for normal cells were significantly higher than the maximum cytotoxic concentrations (MACC) for neoplastic cells. In contrast, for the known antineoplastic drugs MICC and MACC were similar for both normal and neoplastic cells. Thus, cells of benign origin were several times more resistant to the action of T514 than those of neoplastic origin. This selective toxicity towards transformed cells suggested than T514 could be used as an anticancer agent.

On the other hand, Sepulveda Saavedra et al. [16] studied the in vivo effect of T514 using the yeasts *Hansenula polymorpha* and *Candida boidinii* as model organisms. At sublethal doses (2 µg/mL), T514 caused an irreversible and selective damage of peroxisomes: disruptions appeared in the organelle membrane causing leakage of matrix proteins. Damaged peroxisomes were eliminated by macropexophagy (recently, we found that damaged peroxisomes can also be eliminated by micropexophagy [85]). As mentioned earlier, this was the reason by which T514 was renamed peroxisomicine A1 (PA1). PA2 and T544 produce a similar effect on *C. boidinii* [86].

Taking into account the works described above, a hypothesis was raised about the mechanism of action of PA1 as an anticancer agent: since PA1 attacks peroxisomes, and a significant number of malignant cell types contain fewer peroxisomes than normal cells [87,88,89], tumor cells are more easily destroyed than healthy cells [15].

Several works have been carried out in order to deepen in the mechanism of action of PA1 as a potential anticancer agent. Since CAT is a peroxisomal marker enzyme, Moreno-Sepúlveda et al. [90] evaluated the effect in vitro of PA1 on bovine, mouse, and dog liver CAT activity. PA1 inhibited the activity of CAT from the three animal sources in a non-competitive way. PA2, T496, T544, and T510 also inhibited the bovine CAT. It was speculated the existence of a specific inhibitory effect of PA1 on CAT, since this compound was not able to inhibit the activity of peroxidase, fumarase, and cytochrome oxidase.

Later, the effect of PA1 on liver CAT in tissue fragments (in situ) and in mice poisoned intraperitoneally with isolated PA1 (in vivo) was reported. Results demonstrated that PA1 is not able to inhibit CAT activity in both situations. However, morphometric analysis showed that PA1 produce a decrease in the number of hepatic peroxisomes [91].

In rats treated intraperitoneally with isolated PA1, morphometric analysis also demonstrated a significant decrease in the number of liver peroxisomes [92].

In the experiments with yeasts described above in which peroxisomes were damaged by PA1, PA2, and T544 [16,86], inhibition of CAT was also not observed.

Taken together, results of these studies suggested that PA1 causes a direct damage to peroxisomes, and that CAT is not directly involved in the effect of PA1 on malignant cells.

Distribution of PA1 showed to be homogeneous on peroxisomal, mitochondrial, microsomal, and nuclear subcellular fractions prepared from rat liver treated with an acute dose of PA1 [93]. Therefore, interaction of PA1 with other cellular components has been analyzed.

The interaction of PA1 with DNA, and its inhibition on topoisomerase I and II, were examined using complementary biophysical and biochemical methods. The results showed that PA1 has practically no interaction with DNA. It also was found that PA1 inhibit topoisomerase II but not topoisomerase I. In order to assess the cytotoxic potential of PA1 and its effect on the cell cycle, two human leukemia cell lines, HL-60 and HL-60/MX2, were used. HL-60/MX2 cells have reduced topoisomerase II activity. Cell growth inhibition assays revealed a marked cytotoxic effect of PA1 on the HL-60 line, whereas HL-60/MX2 cells showed a reduced sensibility. Likewise, changes in the cell cycle were more pronounced in HL-60 cells than in HL-60/MX2 cells. These changes included the apparition of cells with a DNA content that was less than that of G_1_ cells. Since these kinds of cells are considered apoptotic, activation of the apoptotic machinery by PA1was investigated. Flow cytometry and Western blot analyses revealed that PA1 induced apoptosis in these leukemic cells. Collectively, the results suggested that inhibition of topoisomerase II plays a role in the mechanism of action of PA1 [94].

Velazco et al. [95] analyzed the cytostatic and cytotoxic capacity, as well as the genotoxicity potential of PA1 using the lymphocyte culture system. Mitotic index (MI) and lymphocyte proliferation kinetics (LPK) were used as parameters to evaluate cytostatic and cytotoxic capacity. For both parameters, a concentration-related inhibition was obtained, indicating a toxic effect. The mean frequency of sister chromatid exchanges (SCE) and the frequency of chromosomal aberrations (CA) were analyzed in order to evaluate genotoxicity. PA1 did not cause SCE and neither induced CA. Thus, PA1 stopped active proliferating cells without causing genotoxic damage.

Vargas-Zapata et al. [96] isolated two strains of *H. polymorpha* affected in the utilization of methanol as sole source of carbon and energy after their treatment with PA1. One of those strains was functionally complemented by means of transformation with a genomic library coming from *H. polymorpha*. The authors attributed these findings to the interaction of PA1 with yeast DNA, which had a mutagenic effect. Considering this work and those described in the previous two paragraphs [94,95], it is clear that more research is needed to clarify whether there is an interaction of PA1 with DNA, and the effects that this interaction would have.

Another controversy that exists concerns the mode of cell death by which PA1 would kill cancer cells. In a study, seven malignant cell lines (Jurkat, Hela, MCF-7, HT29, HepG2, HEK293, and 3T3SV2) were induced to die by PA1. In contrast, PA1 did not induce any significant increase of death in the non-tumorigenic cell lines Rat2 and BALB/3T3. Fluorescence microscopy with Hoechst 33,342 staining revealed chromatin condensation and nuclear fragmentation, characteristics of apoptosis, in the cells affected by PA1. Flow cytometry revealed the presence of cells with less than 2C DNA, another hallmark of apoptosis. This technique also demonstrated a significant accumulation of cells in G_2_/M phases of cell cycle. These results indicated that PA1 inhibits proliferation and induces apoptosis in malignant cells in vitro [97]. Previously, another study also found that PA1 produces alterations in the cell cycle and induces apoptosis in neoplastic cells in vitro (see above, reference [94]).

TC-1 tumor cells were implanted subcutaneously into the hind limb of mice. The treatment group received PA1 injected intraperitoneally at days 2, 4, 6, and 8 post-implantation. Tissue samples from the implantation site and from liver, lungs, and kidneys were collected at day 10 after TC-1 cells were implanted; at that time, a tumor cannot yet be detected. Samples were processed for light and electron microscopy, and by TUNEL assay to detect apoptosis. Analysis of the implantation site by light microscopy revealed characteristics of necrosis in TC-1 cells, such as the presence of pyknotic nuclei. Morphological characteristics of necrosis were also found by electron microscopy, since tumor cells showed pyknotic nuclei with rupture of the nuclear envelope, swollen mitochondria with loss of matrix, and rupture of the plasma membrane and leakage of cytoplasm. TUNEL reaction was negative. Control group, in which PA1 was not administered, showed integrity of TC-1 cells. Liver, lungs, and kidneys showed no alterations in both groups. These results indicate that, in vivo, PA1 causes the death of malignant cells by necrosis, and that this toxicity is selective, since liver, lungs, and kidneys showed no alterations. According to the authors of this work, the differences between this and in vitro studies, in which cancer cells die by apoptosis, could be due to the concentration of PA1 that reaches the cells, which could be higher in vitro than in vivo. Regarding selectivity, the difference with other studies in which several organs were damaged by PA1 was attributed to the use of lower doses of PA1 in this study, and also to the fact that doses were administered early after cell implantation [98]. Interestingly, TC-1 cells are resistant to the action of PA1 when they are adjacent or into the sarcoplasm of muscle fibers. The existence of different subpopulations of TC-1 cells could be the explanation for this resistance [99].

On the other hand, the metabolism of PA1 has also been analyzed. The metabolism of this compound was evaluated in vitro in rat and monkey liver microsomes, and in rat and human primary cultured hepatocytes. Microsomes and hepatocytes were incubated with PA1 and the samples were analyzed using HPLC and UV spectra. Two major metabolites (called M1 and M2) were found and isolated. Afterwards, Chang liver and Hep G2 cells were exposed to PA1 and metabolites M1 and M2. The metabolites were less toxic than PA1 and did not show the same selective cytotoxicity on tumor cells as PA1. These data indicate that M1 and M2 are not involved in the selective effect of PA1 [100].

Since the pharmacology and pharmacokinetics of a drug depend on the degree to which it bounds to plasma proteins, the interaction of PA1 with human serum albumin (HSA) and bovine serum albumin (BSA) was investigated using spectrophotometric methods. Results indicated that PA1 binds to both HAS and BSA at physiological pH; binding is stronger with the former. From titration analysis of HAS or BSA with PA1, it was determined that both albumins have two types of binding sites for PA1. Titrations were performed at four different temperatures, and thermodynamic parameters suggested that binding occurs through hydrogen bonding and hydrophobic interactions [101].

Finally, in a clinical phase I study, patients with recurrent cervical cancer treated with PA1 showed stable disease and increased survival time [102]. Patents for the use of PA1 as a probable anticancer agent have been obtained from the European Patent Office [103], the United States of America [104], Canada [105], Japan [106], Korea [107], and Mexico [108].

In summary, T514 (PA1) has shown selective toxicity in vitro against human cancer cells. More research is needed, but the evidence obtained so far indicates that PA1 could be an effective anticancer agent.

## 5. Conclusions

The main bioactive agents of *Karwinskia* plants have been previously isolated and well characterized, including toxins T496, T514, T516, and T544, and several related compounds, such as peroxisomicine A2, peroxisomicine A3, isoperoxisomicines A1 and A2, toxin T510, and tullidinol B1 and B2. However, there is relatively little information about the mechanisms of action by which these substances exert their detrimental effects. Additionally, literature related to the potential antineoplastic effect of T514 or PA1 is scarce and sometimes contradictory. 

Toxins of *Karwinskia* cause damage mainly to nervous system, liver, lung, kidney, cardiac and skeletal muscles, pancreas, and to embryo and fetus. In the future, research regarding damage provoked by *Karwinskia* toxins must be focused in obtaining easily accessible diagnostic methods and effective treatments. Studies devoted to finding the specific targets of the toxins, as well as those that analyze in depth the metabolism of the latter in the organism, can be very useful. 

On the other hand, T514 has shown selective toxicity in vitro against human cancer cells. T514 causes an irreversible and selective damage of peroxisomes (for this reason it was renamed peroxisomicine A1 or PA1) and since a significant number of malignant cell types contain fewer peroxisomes than normal cells, tumor cells could be more easily destroyed than healthy cells. As a perspective, analyzes involving animal cancer models, and the performance of more clinical trials, are necessary to know the true therapeutic value of PA1. Despite these limitations, the evidence obtained so far indicates that PA1 could be an effective anticancer agent.

## Figures and Tables

**Table 1:** Species of *Karwinskia* [2,3].

*K. angustata* Borhidi & O. Muñiz	*K. orbiculata* (Britton & P. Wilson) Urb.
*K. bicolor* (Britton & P. Wilson) Urb.	*K. parvifolia* Rose
*K. calderonii* Standl.	*K. pluvialis* A. Pool
*K. caloneura* Urb.	*K. potrerilloana* (Borhidi & O. Muñiz) Borhidi
*K. colombiana* Dugand & M.C. Johnst.	*K. rocana* (Britton & P. Wilson) Urb.
*K. humboldtiana* (Willd. ex Schult.) Zucc.	*K. rzedowskii* R. Fern.
*K. johnstonii* R. Fern.	*K. sessilifolia* Schltdl.
*K. latifolia* Standl.	*K. subcordata* Schltdl.
*K. microphylla* Suess.	*K. tehuacana* R. Fern. & N. Waksman
*K. mollis* Schltdl.	*K. umbellata* (Cav.) Schltdl.
*K. oblongifolia* (Britton & P. Wilson) Urb.	*K. venturae* R. Fern.

## Abbreviations

BSA	Bovine Serum Albumin
CA	Chromosomal Aberrations
CAT	Catalase
CD	Circular Dichroism
CNS	Central Nervous System
CR	Crown-Rump
DCFDA	Dichlorofluorescein Diacetate
FENa	Fractional Excretion of Sodium
FF	Filtration Fraction
GFR	Glomerular Filtration Rate
HAS	Human Serum Albumin
HMBC	Heteronuclear Multiple Bond Coherence
HMQC	Heteronuclear Multiple Quantum Coherence
HPLC	High Performance Liquid Chromatography
LDH	Lactate Dehydrogenase
LPK	Lymphocyte Proliferation Kinetics
MACC	Maximum Cytotoxic Concentrations
MI	Mitotic Index
MICC	Minimum Cytotoxic Concentrations
MTT	3-[4,5-Dimethylthiazol-2-yl]-2,5-dephenyltetrazolium bromide
NMR	Nuclear Magnetic Resonance
NR	Neutral Red
PA1	Peroxisomicine A1
PA2	Peroxisomicine A2
PA3	Peroxisomicine A3
PCNA	Proliferating Cell Nuclear Antigen
PNS	Peripheral Nervous System
ROS	Reactive Oxygen Species
RPF	Renal Plasma Flow
SCE	Sister Chromatid Exchanges
SOD	Superoxide Dismutase
TLC	Thin-Layer Chromatography
TUNEL	Terminal Deoxynucleotidyl Transferase dUTP Nick End Labeling
UV	Ultraviolet
Vis	Visible
